# Silent myocardial ischemia in prediabetics in relation to insulin resistance

**DOI:** 10.4103/0975-3583.70903

**Published:** 2010

**Authors:** Gamela Nasr, Hamdy Sliem

**Affiliations:** *Department of Cardiology Suez Canal University, Ismailia, Egypt*; 1 *Department of Internal Medicine, Suez Canal University, Ismailia, Egypt*

**Keywords:** Insulin resistance, prediabetes, single photon emission computed tomography

## Abstract

**Background:**

Myocardial perfusion imaging (MPI) is a powerful diagnostic and prognostic tool for evaluating coronary artery disease (CAD). Several studies have shown Type 2 diabetics are at increased risk for having CAD. In addition, insulin resistance is generally considered to be of major importance in the pathophysiology of Type 2 diabetes mellitus. However, the area of screening prediabetics for CAD remains unclear. Given that glucose intolerance and insulin resistance precede the development of overt diabetes, these factors would be associated with CAD.

**Aim:**

This study was designed to evaluate the state of myocardial perfusion in prediabetic adults detected by single photon emission computed tomography (SPECT) in relation to insulin resistance.

**Patients and Methods:**

A descriptive study was performed. Out of 113 consecutive prediabetic adults, 32 had insulin resistance (Group A) and 81 had insulin sensitivity (Group B). All were subjected to full medical history and clinical examination including blood pressure, waist circumference, and body mass index. Biochemical studies including lipids profile, fasting blood glucose, and homeostasis model assessments (HOMA) test. Exercise treadmill technetium (99mTC) sestamibi SPECT scintigraphy were done for assessment of myocardial perfusion assessed by summed difference score as well as occurrence of transient left ventricular dilatation.

**Results:**

Significant increase in summed difference score as well as transient left ventricular dilatation was observed in Group A than Group B. It is correlated with insulin resistance, and the correlation appears to be independent of glucose tolerance status and obesity. Similar correlations were observed with age, triglycerides, and waist circumference.

**Conclusion:**

Prediabetics have myocardial perfusion defects which represent a pattern of cardiovascular risk. These are predominantly observed in prediabetics with increased HOMA IR and visceral obesity independent of glucose levels.

## INTRODUCTION

Prediabetes broadly refers to an intermediate stage between completely normal glucose levels and the clinical entity of Type 2 diabetes. Diabetes mellitus, hypertension, dyslipidemia, obesity, and aging are associated with high risk of cardiovascular disease (CVD) as well as other clinical conditions[1]

The mechanisms through which cardiovascular risk is increased are partially understood. Myocardial perfusion imaging (MPI) is a powerful diagnostic and prognostic tool for evaluating coronary artery disease (CAD). Prognostic indicators on MPI include the severity of both fixed and reversible perfusion defects (myocardial infarction and ischemia),[2] reduced left ventricular function after stress,[3,4] and dilation of the left ventricle after stress.[5–7] Given that glucose intolerance and insulin resistance precede the development of overt diabetes, these factors would be associated with ischemic heart disease and perfusion defects. To investigate this hypothesis, the current study was undertaken to evaluate the state of myocardial perfusion in prediabetic adults in relation to insulin resistance. Stress single-photon emission computed tomographic myocardial perfusion imaging (SPECT) performed with technetium (Tc)-99m–labeled radiopharmaceuticals is widely used for diagnosing CAD and assessing patient risk. Patients determined to have a normal SPECT on the basis of stress imaging alone have a similar mortality rate as those who have a normal SPECT on the basis of evaluation of both stress and rest images.[8]

## PATIENTS AND METHODS

### Study population

A descriptive study enrolling 113 consecutive adults with prediabetes with an age range of 40–50 years was carried out. All were recruited from the outpatient diabetes and general medicine clinics of Suez Canal University Hospital from May 2007 to October 2009. Thirty-two adults had insulin resistance (Group A) and 81 had insulin sensitivity (Group B). Exclusion criteria included hypertension, diabetes mellitus, chronic kidney disease, CVD, severe obesity (body mass index > 40 kg/m^2^), heavy smokers, and aged adults (over 60 years old). All groups were subjected to full medical history and clinical examination including blood pressure (BP), body mass index (BMI), systemic examination, and biochemical studies. Anthropometric measures were weight in kilogram, height in centimeter, body mass index, and waist circumference. Body mass index was calculated as weight/height2 (kg/m^2^) and was used as an estimate of overall adiposity. Normal weight, overweight, and obesity were defined as a BMI less than 25, 25–29.9, and 30 or higher, respectively.[8] Waist circumference, a validated estimate of visceral adiposity, was measured to the nearest 0.5 cm. Central obesity is defined as waist circumference > 102 cm in males and >88 cm in females.[9]

Diabetes was diagnosed according to World Health Organization (WHO) criteria. Blood is drawn after fasting for 8 h. A fasting blood sugar level below 100 mg/dL is considered normal. A fasting blood sugar level between 100 and 126 mg/dL confirms the presence of prediabetes, and more than 126 mg/dL confirms the presence of diabetes in two separate occasions.[10] Homeostasis model of insulin resistance (HOMA IR) was used as a measure of insulin resistance. It was assessed according to the level of fasting glucose and insulin which were measured with a dextran–charcoal radioimmunoassay. Serum intact proinsulin was measured by using a highly specific, 2-site monoclonal antibody-based immunoradiometric assay:[10,11]

The formula for the HOMA IR model is as follows:

$$\;\mathrm{HOMA}\;\mathrm{IR}\;=\;\left(\mathrm{Fasting}\;\mathrm{insulin},\;\mathrm{mU}/\mathrm{mL}\;\times \;\mathrm{Fasting}\;\mathrm{glucose},\;\mathrm{mmol}/L\right)/22.5.$$

Subjects were divided by their insulin resistance status at baseline (HOMA IR above and below median of 3.0) to insulin resistant and insulin sensitive, respectively.[10]

### Exercise treadmill technetium-99m sestamibi SPECT scintigraphy

All prediabetics were scheduled for a treadmill test using an automated programmable treadmill Bruce protocol. Each was examined before the test and 12 lead ECG was recorded and read. Blood pressure was measured by arm-cuff method with an aneroid sphygmomanometer and a stethoscope for auscultation. ST segment depression was measured in millimeter at 80ms from the J point and considered significant when >1 mm from the baseline. Criteria for stopping the test were: target heart rate achieved, occurrence of chest pain or significant ST segment depression, hypertensive or hypotensive blood pressure response or failure of blood pressure to rise >10 mmHg, serious cardiac arrhythmia, and patients request to stop. Both the duration of the exercise and reason for stopping were recorded. The radiotracer 8–10 mCi of Tc99m-sestamibi injected through an intravenous cannula when one of the following occurred: target heart rate achieved, appearance of significant ST segment depression, and significant chest pain. Then, the exercise test was continued for another 45–60 s and then the test terminated. Tomographic imaging was performed 1 h later.

### Interpretation of exercise ECG

An ischemic ST segment response was defined as horizontal or down sloping ST segment depression >1 mm below baseline 80 ms from the J point. Functional capacity measured in metabolic equivalents (or METs, where one MET is 3.5 mL/kg per min of oxygen consumption.

### Reconstruction and interpretation of the technetium- 99m-sestamibi

Imaging was acquired on rotating gamma camera (Anger Scintillation Gamma Camera, General Electric—Maxi Camera 400 AC) equipped with a general all-purpose collimator interfaced to a dedicated computer system. Acquisition in 32 stops each for 40 s in 64 × 64 matrix imaging was performed through three windows. Oblique tomograms are generated in vertical and horizontal long axis and short axis orientation using an operator-dependent computerized algorism. The tomograms then normalized so the hottest pixel for each orientation for both exercise and delayed tomograms is set to uniform intensity. The myocardium segments usually interpreted for the presence of perfusion abnormalities.

The summed stress and rest scores were obtained by adding the scores of the 20 segments of the respective images.[12] The sum of the differences between each of the 20 segments from these images was defined as the summed difference score, representing the amount of ischemia. Each of these variables incorporates the extent and severity of perfusion. The summed difference score was converted to percent myocardium ischemia according to a published ≥ algorithm. “Ischemic” and “moderately to severely ischemic” where MPS studies were defined by ≥5 and ≥10% of the myocardium, respectively.[12] Images were also interpreted visually and by consensus for the presence or absence of transient ischemic dilation by two experienced observers who were unaware of the clinical findings.

### Ethical consideration

Informed consent was obtained from all the adults. The aim and the value of the work were explained in a simplified manner for them. There was no harm inflicted on them. On the contrary, all had benefits of the follow-up and the final results of the study. In addition, it was a descriptive study in prediabetics as it is unethical to perform the test in otherwise normal non-risky controls.

### Statistical analysis

Data were presented in terms of mean and standard deviation (SD) of the mean, and percentages. Statistical analysis was carried out by a computer program (SPSS Ver. 11). ANOVA and correlation tests were used to evaluate the results between the three groups. *P* value was set at <0.05 for statistically significant results and <0.0001 for highly significant results. Logistic regression analysis was used to assess odds ratio.

## RESULTS

Descriptive baseline characteristics of 113 (60 males and 53 females) prediabetic adults, mean age 43.1 years, with and without insulin resistance are shown in Table 1. Of them, 36.2% of the prediabetic adults had insulin resistance and 63.8% had insulin sensitive. Except for the waist circumference, all the mentioned parameters were nearly similar in both male and female prediabetic adults.

**Table 1:** Clinical and biochemical studies of prediabetics

Variable	Prediabetic adults (case), N = 113			*P* value		
	All, N = 113	Group A, N = 32	Group B, N = 81	P*	P**	P***
Age	45.1 +6.2	44.9 ±7.1	45.3 ±5.9	n.s	n.s	n.s
SBP	122.2 ±9.2	120.9 ± 7.9	120.1 ±9.5	n.s	n.s	n.s
DBP	73.9 ± 6.9	74.9 ± 6.5	73.5 ±7.1	n.s	n.s	n.s
BMI	28.4 ± 5.2	28.5 ±5.2	28.4 ±5.1	n.s	n.s	n.s
Waist circumference	103 ± 17.2	107 ± 11.6	102.5 ± 18.2	n.s	n.s	n.s
Total lipids	491 ±31.9	479 ± 26.7	499.7 ± 36.4	n.s	n.s	n.s
Triglycerides	187.7 ± 12.4	187 ±14.2	188.5 + 11.9	n.s	n.s	n.s
FBG	114.5 ±4.8	114.8 ±4.6	115.7 ±4.9	<0.01	<0.01	n.s
HOMAIR	3.7 ±2.5	7.5 ±0.7	2.2 ± 0.4	<0.01	<0.01	n.s
Sum dif. score	3.11 ±1.31	11.87 ±3.91	2.89 ±172	<0.001	<0.01	n.s
Trans isch. dilation	11/113 (9.7%)	7/32 (21.9%)	4/81 (4.9%)	<0.01	<0.0I	n.s

Group A = Prediabetic adult with insulin resistance; Group B = prediabetic adult with insulin sensitivity; BMI = body mass index; SBP = systolic blood pressure; DBP = diastolic BP; N = number of cases; n.s = nonsignificant; FBG = fasting blood glucose; Sum dif score = Summed difference score; Trans isch dilatation = transient ischemic dilation.

* P = Comparison between Group A and Group B.

** P = Comparison between prediabetic adults and Group A.

*** P = Comparison between all prediabetic adults and Group B.

Correlation between summed difference score and different variables of insulin-resistant prediabetic group (Group A) is shown in Table 2. There was statistically significant coefficient correlation with HOMA IR, waist circumference, triglycerides, and age. No correlation was observed with fasting glucose, BMI, blood pressure, and total lipids. Similarly, no correlations were found with overall prediabetic population variables. The visual consensus method of determining the presence or absence of transient ischemic dilation resulted in classification of 11 of 113 persons as having transient ischemic dilation (9.7%). In the insulin-resistant group, it was 7/32 (21.9%) and lastly in the insulin-sensitive group it was 4/81 (4.9%).

**Table 2:** Correlation between summed difference score and different variables in insulin-resistant prediabetics

Variable	*r* value	*P* value	Variable	*r* value	*P* value
Age	0.84	<0.01	Total lipids	0.44	n.s.
Total cholesterol	0.11	n.s.	Systolic blood pressure	0.38	n.s.
Low density lipoprotein	0.24	n.s.	Diastolic blood pressure	0.36	n.s.
Triglyceride	0.79	<0.01	High density lipoprotein	0.24	n.s.
HOMA IR	0.92	<0.001	Fasting blood glucose	0.18	n.s.
Waist circumference	0.87	<0.01	Body mass index	0.18	n.s.

n.s. = Nonsignificant.

## DISCUSSION

It is well known that Type 2 diabetes mellitus is associated with a marked increase in CVD. Increased risk factors for CVD before the onset of Type 2 diabetes have been shown in several populations.[1,13,14] It is not known whether the increased atherogenicity of the prediabetic state is primarily due to increased insulin resistance or impaired blood glucose.[15]

It has been recognized that prediabetic hyperglycemia confers an increased risk for cardiovascular disease (CVD).[16,17] In 1997, the American Diabetes Association (ADA) introduced the concept of impaired fasting glucose (IFG), a prediabetic state initially defined by fasting plasma glucose (FPG) of 110–125mg/dL, in which those afflicted were significantly more likely to develop diabetes.[18–20] The risk of developing CVD was not considered in establishing criteria for IFG. Since the introduction of the concept of IFG, there has been considerable debate regarding where the lower limit should be set to achieve a reasonable balance between sensitivity and specificity for diabetes prediction. In 2003, the ADA lowered its threshold for diagnosis of IFG from 110 mg/dL to 100 mg/dL on the basis of evidence in selected samples that suggested diabetes prediction may be optimized at a lower threshold.[21] The effect of this lowered cut point is that a much larger proportion of the population is now considered to have IFG. Using data from the Third National Health and Nutrition Examination Survey, Benjamin *et al*.[22] found that the prevalence of IFG among adults was estimated to increase from 8.3% to 30.2%.

In the context of current literature, conflicting data exist regarding the effect of nondiabetic fasting hyperglycemia on cardiovascular risk. Although some studies have found that the 1997 IFG definition is associated with significantly increased risk for CVD,[23,24] others have shown no significantly increased risk for CVD with the 1997 IFG definition.[25–27]

The progression from prediabetes to Type 2 diabetes occurs over many years before the development of overt hyperglycemia seen in diabetes.[28,29] However, the state of perfusion in prediabetes has received little attention. We, therefore, studied prediabetic normotensive adults in whom the prevalence of insulin resistance and impaired glucose tolerance is high to test the hypotheses that insulin resistance is associated with myocardial perfusion defects and that this relationship is dependent or independent of glucose tolerance status.

The main finding of this study is that insulin resistance is associated with myocardial perfusion defects and that the association appears to be independent of glucose tolerance status and obesity. Results from this study did not identify an increase in myocardial perfusion defects among individuals with insulin-sensitive prediabetes. Furthermore, no correlation was found between them and fasting glucose measurements. Our data suggest that insulin resistance may be more important in the development of myocardial perfusion defects. The mechanism underlying the relationship between insulin resistance and myocardial perfusion defects is not clear enough. Prediabetes is not only a significant risk factor for progression to Type 2 diabetes, but is also considered a risk factor for macrovascular disease and for retinopathy. Some of this risk may be associated with progression to overt diabetes, but there is still increased risk in individuals who have not yet progressed to diabetes. A meta-analysis of 38 prospective studies, for example, suggests that postchallenge blood glucose levels in the nondiabetic range appear to have a linear relationship with cardiovascular disease risk and a possible threshold risk with FPG of about 100 mg/dL.[30]

The risk of progressing to diabetes depends on the degree of insulin resistance and deficiency of insulin secretion as well as other diabetes risk factors, such as age, family history, overweight/obesity, or history of gestational diabetes.[31,32] Previous studies have shown that visceral fat in young healthy individuals and older adults is associated with increased cardiovascular risk factors.[33] This study confirms this association. Although it is observed that abdominal adiposity as measured by waist circumference was strongly and adversely associated with myocardial perfusion defects, body mass index as a measure of general adiposity was not. In addition, this study found that insulin resistance appears to be more strongly associated with perfusion defects than with measures of obesity. These results suggest that the obesity–perfusion defects relationship may be mediated in part through increasing insulin resistance. In general, adipocytes, in particular from visceral abdominal regions, produce several bioactive peptides which in turn impact on microcirculation.[34,35] However, impact of hypertension upon microcirculation was ameliorated as all subjects were normotensives, such the association was not held. On the other hand, total lipids had insignificant correlation with myocardial perfusion defects. When the components of the lipids were considered separately, perfusion defect showed direct associations only with triglycerides in this study, age was an independent predictor of having perfusion defects. It is known that different arterial segments respond differently to aging.[36]

In conclusion, we have shown that prediabetics have myocardial perfusion defects which represent a pattern of cardiovascular risk, and these changes are predominantly observed in prediabetics with increased HOMA IR and visceral obesity. As the silent ischemic changes in the prediabetic state are limited to subjects with insulin resistance, the use of insulin-sensitizing agents to prevent diabetes could have a beneficial effect on CVD. None of the studied prediabetic adult had symptomatic cardiac disease. The findings of this study revealed that changes can be detected before the appearance of clinically apparent coronary myocardial perfusion defects and may act as a marker for the development of future CVD. It is likely that over the next few years screening for ischemic in asymptomatic prediabetics for perfusion defects will become an increasingly important part of the process of risk assessment, and may possibly also improve the monitoring of therapy.[30] However, these studies are beyond doubt have limitations regarding radiation exposure, cost, and feasibility. Additional studies will be needed to assess the effect of an improvement in insulin sensitivity to improve the myocardial perfusion as well to investigate cost effectiveness of using SPECT in prediabetics.

It is important to note several limitations in this study design, first, the relatively small sample size. Second, this study enrolled a relatively population of older individuals carefully screened to exclude diabetes and hypertension. Further research will be needed to confirm whether these results generalize to middle-aged, more insulin-sensitive individuals and those with hypertension through a large community-based study. Further studies are needed for prevention of Type 2 diabetes and the prediabetic state as well.
